# In-Situ Classification of Highly Deformed Corrugated Board Using Convolution Neural Networks

**DOI:** 10.3390/s24041051

**Published:** 2024-02-06

**Authors:** Maciej Rogalka, Jakub Krzysztof Grabski, Tomasz Garbowski

**Affiliations:** 1 Institute of Applied Mechanics, Poznan University of Technology, 60-965 Poznan, Poland; maciej.rogalka@o2.pl; 2 Department of Biosystems Engineering, Poznan University of Life Sciences, 60-627 Poznan, Poland; tomasz.garbowski@up.poznan.pl

**Keywords:** corrugated board, flute type, cross-section image, convolutional neural network

## Abstract

The extensive use of corrugated board in the packaging industry is attributed to its excellent cushioning, mechanical properties, and environmental benefits like recyclability and biodegradability. The integrity of corrugated board depends on various factors, including its geometric design, paper quality, the number of layers, and environmental conditions such as humidity and temperature. This study introduces an innovative application of convolutional neural networks (CNNs) for analyzing and classifying images of corrugated boards, particularly those with deformations. For this purpose, a special device with advanced imaging capabilities, including a high-resolution camera and image sensor, was developed and used to acquire detailed cross-section images of the corrugated boards. The samples of seven types of corrugated board were studied. The proposed approach involves optimizing CNNs to enhance their classification performance. Despite challenges posed by deformed samples, the methodology demonstrates high accuracy in most cases, though a few samples posed recognition difficulties. The findings of this research are significant for the packaging industry, offering a sophisticated method for quality control and defect detection in corrugated board production. The best classification accuracy obtained achieved more than 99%. This could lead to improved product quality and reduced waste. Additionally, this study paves the way for future research on applying machine learning for material quality assessment, which could have broader implications beyond the packaging sector.

## 1. Introduction

Corrugated board is widely utilized in packaging food products, transporting various goods and other applications. Its main advantages include being lightweight and easy to handle, along with the ability to be printed with custom designs. As a recyclable and biodegradable material, corrugated board is an eco-friendly choice for businesses and consumers. It is a versatile and well-liked material employed in the packaging sector [1,2]. Its structure comprises a ridged sheet, a flute and two smooth linerboards, providing resilience and flexibility.

The flute in the corrugated board is produced by feeding paper through grooving rolls, which create the distinctive ridges and depressions, giving the board its name. The flutes vary in dimensions, with higher flutes offering more strength and cushioning, and lower flutes providing a smoother surface for printing. The outer layers of corrugated board typically made from kraft paper, known for its durability and tear resistance, make it an ideal packaging material.

However, corrugated board can become distorted during production, storage, transportation or use due to factors like temperature and humidity fluctuations, or mechanical loads. There are two types of corrugated board imperfections: global or local. The literature includes studies on these imperfections and their impacts on mechanical properties. Models of large-scale deflections of cardboard have been developed by Beck and Ficherauer [3]. The authors mainly analyzed local imperfections. Nordstrand studied the impact of their size on the compressive strength of boxes made of corrugated board [4]. Later, the same author analyzed the nonlinear buckling of Rhodes and Harvey orthotropic plates to study local imperfections [5]. Lu et al. examined the behavior of the corrugated boards with imperfections during compression to analyze their mechanical properties [6]. An analytical study of double-walled corrugated board during a bending test was proposed by Garbowski and Knitter-Piątkowska [7]. Mrówczyński et al. developed a method to analyze single-walled corrugated board by introducing initial imperfections [8]. Cillie and Coetzee investigated corrugated boards under in-plane compression [9]. The corrugated boards included both local and global imperfections. Recently, Mrówczyński and Garbowski applied the finite element method and representative volumetric element to study the effective stiffness of corrugated board with geometrical imperfections [10].

In the literature, one can find many techniques for testing the corrugated board that boxes are made from. Compressive, tensile, or bursting strength tests are commonly performed to evaluate the mechanical properties of corrugated board. The box compression test (BCT) and the edge crush test (ECT) [11,12] are the best known in the packaging industry. The mechanical strength of paperboard or corrugated cardboard boxes has a strong connection to two distinct in-plane directions of orthotropy. These directions are perpendicular to the main axis of the fluting and parallel to the alignment of the paperboard fibers. One direction is known as the machine direction (MD), which goes parallel to the fibers, and the other is the cross direction (CD), which goes parallel to the fluting. Another approach in assessing the mechanical strength of boxes made from corrugated board is to use analytical formulas, e.g., the very famous McKee formula [13] or its modifications, such as those proposed in [14,15]. On the other hand, one can perform numerical analyses of the corrugated board [16,17,18,19], which is also a common methodology used in much research in this area. The approach presented in this paper can lead to the automatic creation of a 3D model of the real corrugated board structure based on images in future research. Here, we propose a first step towards this.

Analyzing corrugated board using image processing techniques and computer vision is not a common problem addressed in the literature. However, there are some works in this area, mainly those related to designing systems for automatic waste sorting. Transfer learning and model fusion were applied by Liu et al. to propose a new method for garbage classification [20]. Template matching was used by Rahman et al. to classify and sort recyclable waste paper [21]. In terms of corrugated boards, studies have focused on the automatic counting of its layers using image processing techniques. For example, Cebeci applied classical image processing operations for counting corrugated boards [22]. Similarly, Suppitaksakul and Rattakorn employed a machine vision system and image processing techniques for this purpose [23]. Suppitaksakul and Suwannakit proposed a procedure for stitching corrugated board images [24].

A convolutional neural network (CNN) is a deep learning model specifically designed for processing and analyzing visual data, such as images and videos. Inspired by the human brain’s visual cortex, CNNs are highly effective in tasks like image classification, object detection, and other image processing challenges. These networks utilize convolutional layers to automatically detect and extract patterns from input data, which can include various visual features loke edges, shapes, textures, and other characteristics. Convolutional layers employ filters, or kernels, to analyze the input data by performing element-wise multiplication and summation, thus producing a feature map. This process aims to highlight specific characteristics presented in the data.

The study of cross-section geometry and material classification based on images is relatively rare in the literature. However, there are some examples. These studies primarily utilize machine learning techniques, including CNNs. Caputo et al. applied support vector machines to classify materials from images [25] also acquired under various illumination and pose conditions [26], using a pretrained ResNet-50 network architecture. Wyder and Lipson identified the static and dynamic properties of cantilever beams using the CNNs, basing their classification on raw cross-section images [27]. Li et al. explored different deep learning techniques to analyze the geometric features of self-piercing riveting cross-section, with SOLOv2 and U-Net architectures yielding the best results [28]. Ma et al. conducted a study on the geometrical parameters of crushed thin-walled carbon fiber-reinforced polymer tubes cross-sections [29]. Daigo et al. proposed the use of PSPNet to estimate the thickness of steel in heavy melting scrap [30]. The CNN and conditional generation antagonism model were utilized by Liu et al. to predict the cross-sectional shape and damage morphology of self-piercing riveted joints in carbon fiber-reinforced composites and aluminum alloy [31]. Recently, Kato et al. evaluated the internal cracks of timbers using CNNs [32,33]. The optimal thickness of blending composite laminates was determined by Huynh et al. using the CNN and genetic algorithm [34].

In this paper, the authors proposed using CNNs for classifying types of corrugated board. This classification depends on the flute of the board, a feature significantly influencing its mechanical properties. To the best knowledge of the authors, such an approach has not yet been applied to analyze the geometry of the corrugated board cross-section. The study involved analyzing twenty-seven CNN structures. The most effective models were selected for further discussion regarding their accuracy in the final classification process of this innovative approach.

The automatic classification of the corrugated board was previously considered by the authors of [35,36]. However, in the previous approach, classical image processing methods and genetic algorithms were used to identify geometric features of the corrugated board and later to automatically classify its type [37]. In this paper, the CNNs were proposed for the same purpose. This methodology gave much better results than the approach based on the classical image processing techniques and genetic algorithm, even if the sample was significantly deformed. Furthermore, the computation time is much lower. To the best knowledge of the authors, there are no other papers that consider the classification of the corrugated boards based on its images. This can be a first step in the automatization of corrugated board modeling based on their cross-sectional pictures, which can lead to more realistic numerical analyses of these structures with real imperfections.

## 2. Materials and Methods

### 2.1. Corrugated Boards and Their Types

**Figure 1:**
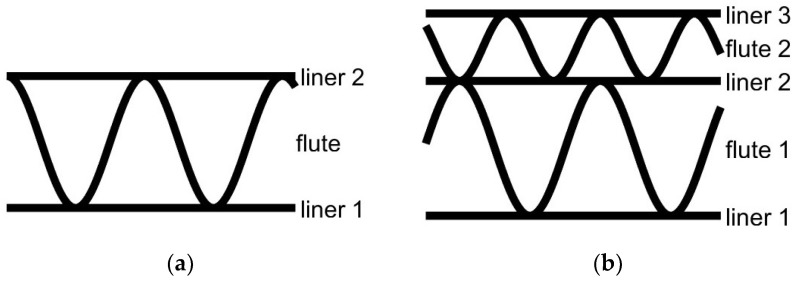
Cross-sections of the corrugated boards for: (**a**) single-walled (3-ply) board; (**b**) double-walled (5-ply) board.

The basic structure of the corrugated board consists of two liners and one flute for a single-wall corrugated board, see Figure 1a. On the other hand, the double-wall corrugated board includes three liners and two flutes—see Figure 1b.

The corrugated boards can be classified based on their geometrical features. In the case of single-walled boards, the most important feature is the height of the flute. Based on this parameter, the most corrugated boards include [35]:

- A—flute with a height of approximately 5 mm;
- B—flute with a height of approximately 3 mm;
- C—flute with a height of approximately 4 mm;
- E—flute with a height of approximately 1.6 mm;
- F—flute with a height of approximately 0.8 mm.

The corrugated boards offer various features of the structures depending on the flute type. For instance, the corrugated board containing a higher flute gives more mechanical strength and cushioning properties, while the smaller flutes provide a smoother surface. Therefore, the former are utilized, for example, for heavy goods protection and transportation, like for furniture, and the latter are useful for detail packaging or packaging for printing purposes. The abovementioned flutes are schematically depicted in Figure 1a.

**Figure 2:**
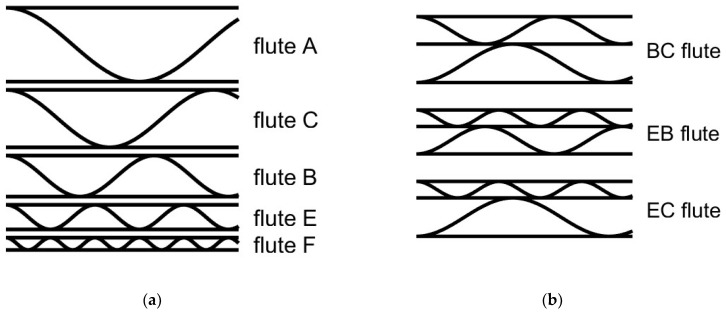
Schematic representations of the corrugated boards commonly available on the market: (**a**) single-walled corrugated boards; (**b**) double-walled corrugated boards.

The benefits resulting from the application of these types of flutes can be merged by combining two types of flutes in double-walled corrugated boards. These can be applied also for improving some specific features of the corrugated boards, for instance, their mechanical strength. The double walls available on the market are often composed of BC (5–7 mm), EB (3.5–5 mm), or EC (4–5.5 mm) flutes [7]. They are schematically depicted in Figure 2b.

In this study, both single- and double-walled corrugated boards are considered. One should notice that in Figure 1 and Figure 2, the ideal structures are presented. In the real situation, where the cross-section is obtained from the image, the structure is deformed, which makes the automatic classification of the original structure more difficult. Figure 2 shows schematic representations of the corrugated board types considered in this study, which are commonly available on the market.

### 2.2. Data Acquition

**Figure 3:**
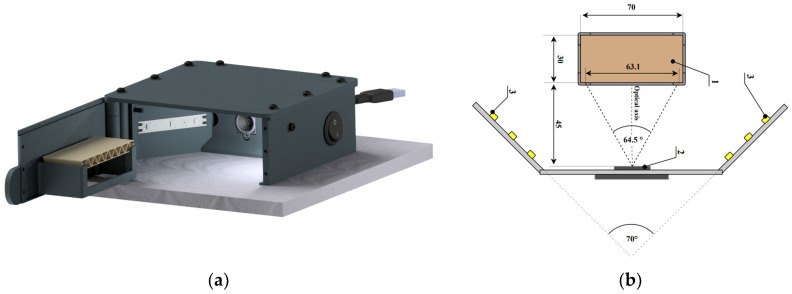
Device for corrugated board image acquisition: (**a**) visualization of the device; (**b**) layout diagram of the most important components of the device (1—corrugated board sample; 2—camera; 3—LED strip (all dimensions in this picture are given in mm)).

A specialized device was developed and built for capturing images of the corrugated board’s cross-section. This device ensures consistent conditions for recording the cross-sectional images of the samples. As depicted in Figure 3a, the device includes a sample holder on the door, which can be opened to easily mount the sample. Neodymium magnets in the door frame secure the door’s closure and prevent unintended opening. The camera, affixed to the device’s frame, is positioned to aim its optical axis straight at the sample’s surface (see Figure 3b). The surface is evenly lit by two LED strips, each with 4.8 W/m power, placed on the partition wall. The light is manually operated with a bistable key switch. External connections from the device include a power cable for the lighting and a USB cable for image transfer to a computer. The device’s components were created using 3D printing.

For image capture, the system utilizes an ArduCam B0197 camera, featuring autofocus and an 8 MPx Sony IMX179 (1/3.2″) image sensor. Images captured by the camera are saved in JPEG format with a maximum resolution of 3264 × 2448 pixels.

### 2.3. Dataset

The samples used for the presented research were obtained from FEMAT [38], a company that specializes in the strength analysis of corrugated board and with whom many local board producers have established collaborative relationship. Some of the samples were deliberately deformed by creasing, allowing for a detailed examination of the visual systems’ response to such deformations. This partnership with FEMAT not only facilitated access to high-quality, relevant samples but also ensured that the experiments were grounded in real-world applications of corrugated board analysis.

**Figure 4:**
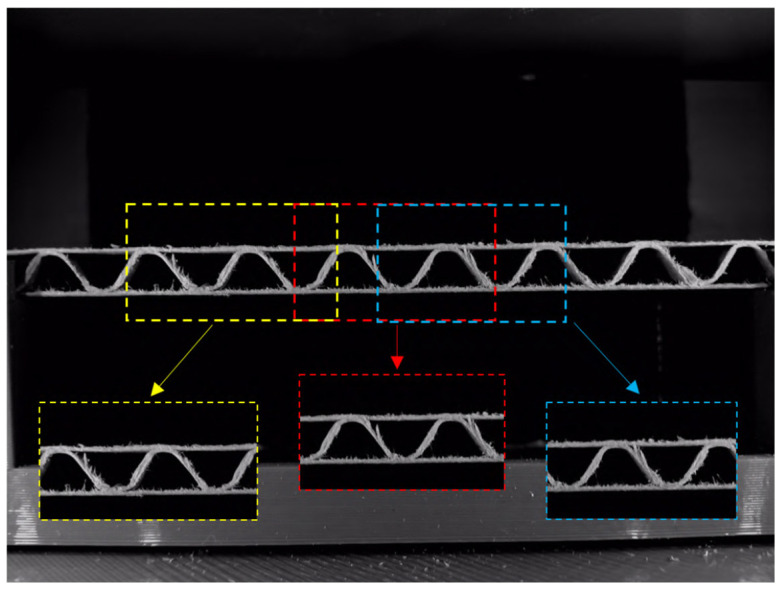
Example of acquired image with smaller images (presented yellow, red, and blue frames) of dimension used to generate the dataset applied in this study.

Using the equipment presented in the previous section, a total number of 646 samples were acquired, and as the results, images of their cross-sections were obtained. Some samples were deformed manually in a random way or using a creasing machine. Within the group of samples with the same flute type, the numbers for non-deformed, manually deformed and creasing machine deformed were the same. The number of images representing the corrugated boards with the specific types of flutes is given in Table 1. In order to unify the number of images in the database for each flute type, smaller images with dimensions of 800 × 800 px were cut out from a large image with dimensions of 3264 × 2448 px. The smaller images were cut out at a random distance from the left image boundary. The idea of creating smaller images from the originally acquired image is schematically presented in Figure 4.

**Figure 5:**
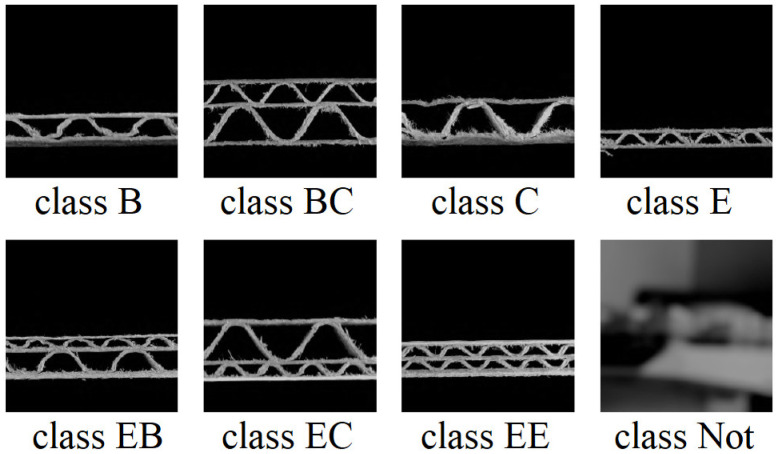
Eight classes of the corrugated board types considered in this study.

After the process of generating the images, in the manner presented in Figure 4, seven classes were obtained (classes B, BC, C, E, EB, EC, and EE). However, the classifiers studied in this paper should also be used to recognize images with no sample of the corrugated board. Therefore, images with an additional class (class Not) were generated to represent situations in which the sample is not present in the acquisition device, the door of the device is not closed, etc. If there were no additional class, the model would give an answer within the seven classes it knows, which would be an obvious mistake. Examples of all eight classes are presented in Figure 5.

The final number of images representing each class and form of sample (non-deformed, deformed manually or deformed using creasing machine) is summarized in Table 2.

Four divisions of the dataset (*dataset1*, *dataset2*, *dataset3*, *dataset4*) ae randomly generated. However, it was ensured that all data types containing 70 images were uniformly represented in training, validation and test sets within each dataset division. Each data type contained 70 images (for instance, the images presenting the manually deformed corrugated board with flute B):

- 49 images (70%) were randomly selected for the training set;
- 10 images (14.3%) were randomly selected for the validation set;
- 11 images (15.7%) were randomly selected for the test set.

For class Not, 147 images were used in the training set, 32 in the validation, and 31 in the test set. This resulted in a training set containing 1176, a validation set including 240, and a test set with 264 images in total for each dataset (*dataset1*, *dataset2*, *dataset3*, *dataset4*).

**Figure 6:**
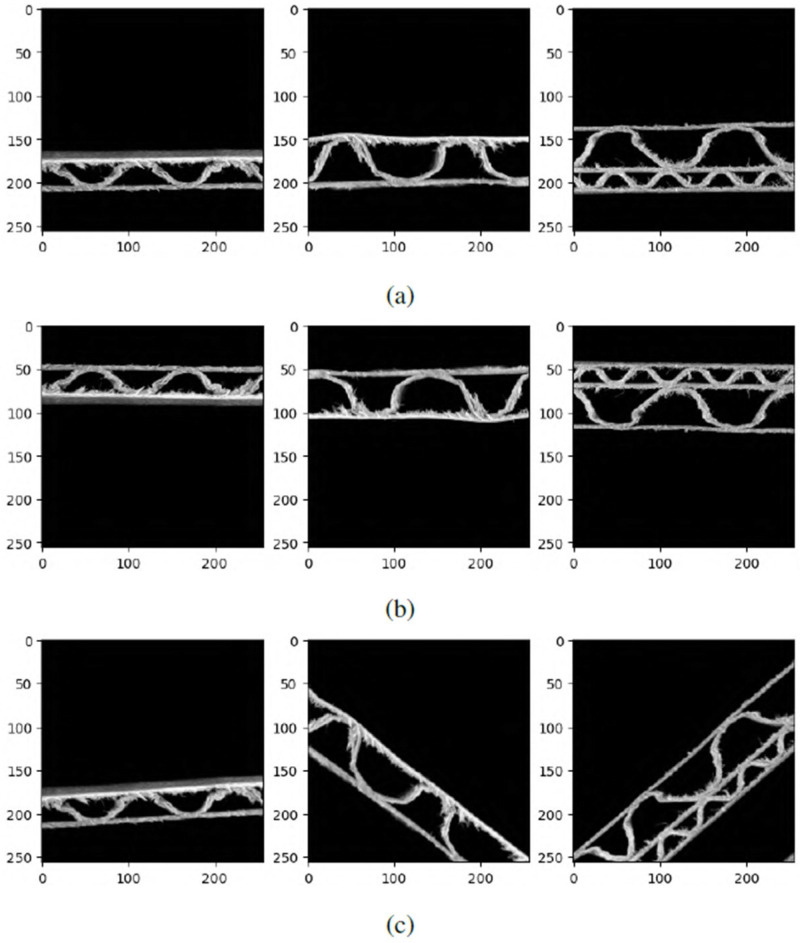
Data augmentation examples: (**a**) original images; (**b**) images randomly flipped vertically or horizontally; (**c**) rotation by a random angle. The dimensions in both directions are expressed in pixels.

Additionally, data augmentation was applied in the current study. This means that the images were randomly rotated by a random angle, or flipped horizontally or vertically. This process ensures that the trained model will also be able to classify images that differ from the images used in the generated dataset. The images do not always need to be acquired horizontally as in the acquisition device used in this study. Examples of data augmentation from the generated dataset are presented in Figure 6.

### 2.4. Convolutional Neural Network

**Figure 7:**
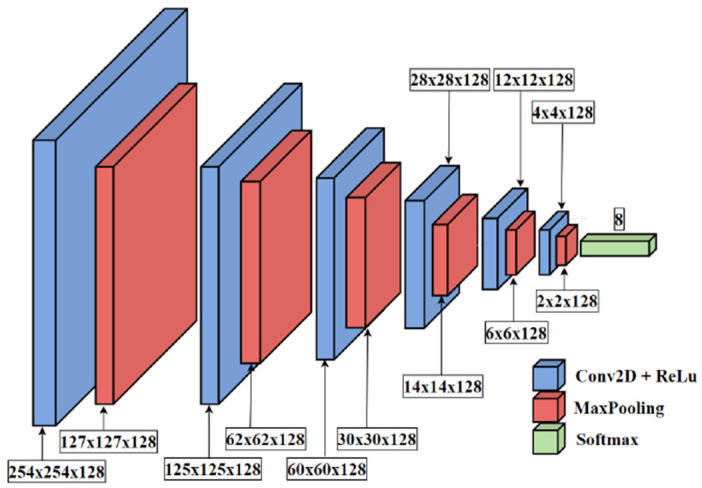
Example of the convolutional neural network structure applied in this study.

An example of the CNN structure studied in this paper is presented in Figure 7. This represents the structure for which the best results were obtained. However, in this study some parameters of this structure were changed: number of convolutional layers, layer size (number of filters), and number of dense layers. The structure shown in Figure 7 consists of six convolutional layers with ReLu activation functions (blue box in Figure 7). After each convolution, one can observe the MaxPooling layer (red box), which reduces the size of the feature map. After this layer, the Flatten layer (to transform a tensor to a vector) and the Dense layers, with a number of neurons equal to the number of filters and ReLu activation functions, can be applied. However, the structure yielding the best results did not include these elements. Here, a Dropout layer is applied to avoid overfitting. In the end, one Dense layer is applied with the eight neurons and Sofmax activation functions. Each neuron in this layer is responsible for the classification of other flute types, as presented in Figure 5.

During the training process, the ADAM (Adaptive Moment Estimation) optimizer was utilized. The sparse categorical cross-entropy was chosen as the loss function and the classification accuracy was adopted as the metric. The training process was always limited to a number of epochs equal to 35, and a batch size of 32 was utilized.

## 3. Results

In order to find the most accurate and universal convolutional neural network architecture, trainings and tests of the CNN were performed by changing the following hyperparameters of the CNN:

- Number of convolutional layers—4, 5 or 6;
- Layer size (number of filters)—32, 64 or 128;
- Number of dense layers—0, 1 or 2.

Combinations of the abovementioned parameters result in 27 various structures of the CNNs.

The training and evaluation process of all 27 types of models was carried out four times on the basis of four previously prepared test datasets: *dataset1*, *dataset2*, *dataset3*, and *dataset4*. This is a kind of cross-validation, which is intended to provide an objective assessment of the suitability of networks with different structures for the issue under consideration. Training and evaluating models with the same hyperparameters for several different arrangements of divisions into training and testing sets ensures the greater reliability of the obtained results.

The results for all the trained models, in the form of accuracies obtained on test sets from four different datasets, are presented in Table 3. The highest average accuracy of 99.04% was obtained for the convolutional neural network *6conv_0dense_128nodes*. This is a neural network with six convolutional layers with 128 filters in each layer and no dense layer. When analyzing Table 3, one can notice that the best results were obtained for architectures with five and six convolutional layers, and with or without one dense layer.

**Figure 8:**
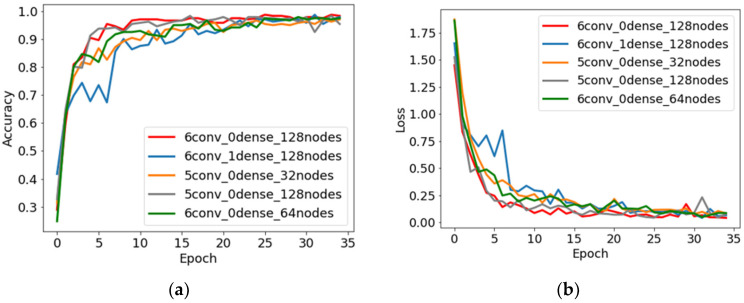

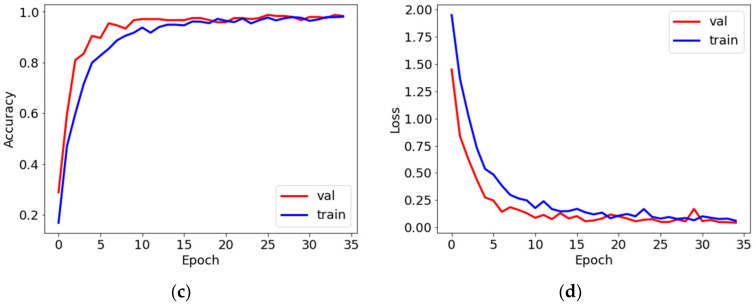
Training curves: accuracy vs. number of epochs (**a**) and loss vs. number of epochs (**b**) plots for six structures with the best results obtained; accuracy vs. number of epochs (**c**) and loss vs. number of epochs (**d**) for training and validation datasets for the convolutional neural network structure with the best results obtained.

Examples of training curves of models with the best accuracy presented in Table 3 are shown in Figure 8. The models, after about 25 epochs, achieved a relatively high training accuracy of about 97%. The values of the training and validation loss also provide information about how well the network copes with a given issue. An increase in the value of the loss on the validation set for subsequent iterations could indicate, for example, the overtraining of the network. In the case presented in Figure 8d, an initial decrease in the loss value is visible, which then stabilizes with the continuation of the training process. Due to dropout regularization, the accuracy and loss for the validation sets achieved better values than for the training sets.

**Figure 9:**
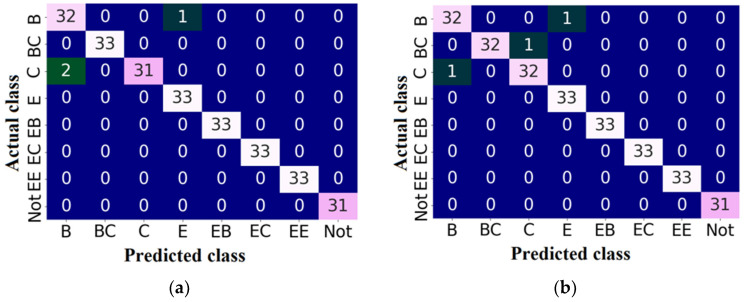

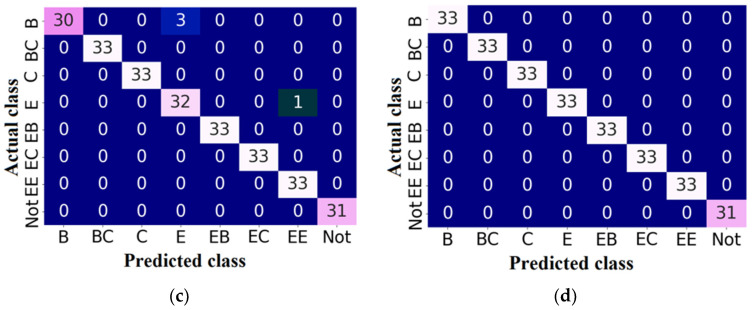
Confusion matrices for the convolutional neural network *6conv_0dense_128nodes* obtained in the training and testing process based on: (**a**) *dataset1*; (**b**) *dataset2*; (**c**) *dataset3*; (**d**) *dataset4*.

Figure 9 shows the confusion matrices obtained for all four versions of the trained model with the best convolutional neural network architecture *6conv_0dense_128nodes*. In these tables, the rows indicate the actual class, and the columns indicate the predicted class. The diagonal represents the number of correctly classified examples for all eight classes included in the datasets. All versions of the model incorrectly predicted the class for 10 images, while in 1038 cases, the flute class was correctly classified. It is visible that if the trained models make an error, they have the highest tendency to misclassify samples with flutes B and C. The confusion matrices indicate that each time, the incorrectly recognized cardboard samples with flutes B and C were assigned to the class representing a lower wave (flutes E and B, respectively). This is most likely directly related to the crease, as a result of which the overall wave thickness and the fluting height are reduced.

**Figure 10:**
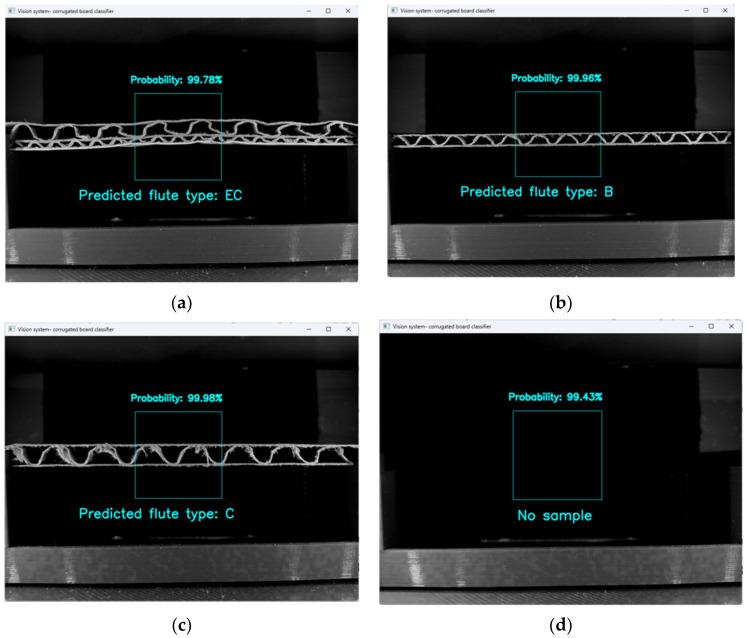
Examples of classification results for: (**a**) flute BC; (**b**) flute B; (**c**) flute C; (**d**) no sample case.

Examples of correctly classified samples are presented in Figure 10.

## 4. Discussion

**Figure 11:**
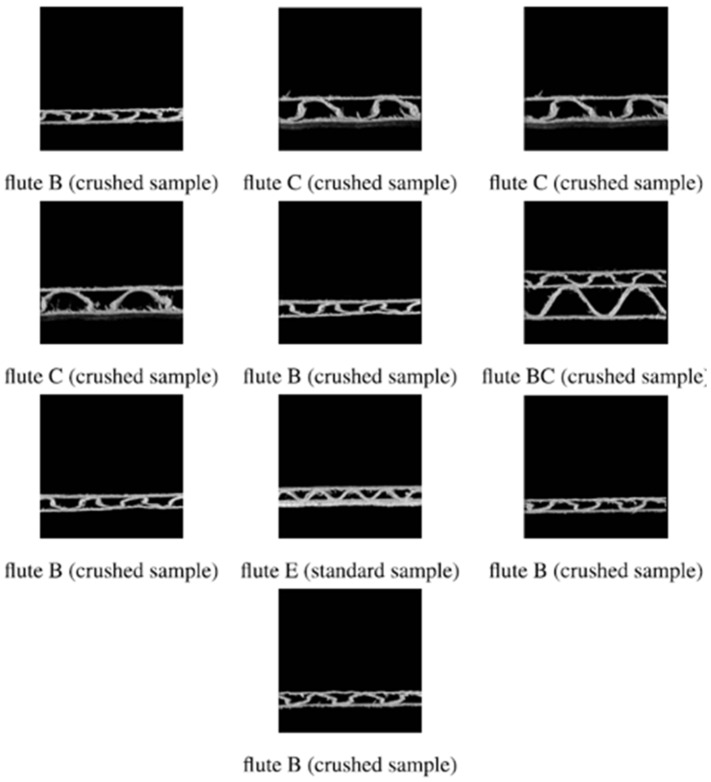
All images from the test datasets incorrectly classified using the convolutional neural network *6conv_0dense_128nodes*.

In this section, the analysis of incorrectly recognized corrugated cardboard samples is performed for the convolutional neural network architecture *6conv_0dense_128nodes*, for which the best accuracy was obtained. In the confusion matrices (Figure 9), one can notice that for *dataset1* and *dataset2*, there were three incorrectly classified samples, for *dataset3* there were four incorrectly classified samples, and for *dataset4*, all the samples were correctly classified. This resulted in 10 cases of incorrect classification in total. Figure 11 shows all these examples. One can see that 9 out of 10 incorrectly classified images showed crushed samples. Regarding the number of layers, 9 of 10 images show three-layer samples. As shown in the results section, if the trained models made errors, they tended to classify images to a class with a smaller wavelength than the one presented (class C classified to class B, class B to class E). It is also worth analyzing the samples presented in the images for other types of imperfections than creasing. None of the samples presented have an exceptionally high number of paper fibers. There are also no inclined samples or samples with delamination. Therefore, the degree of creasing of the sample has the most significant impact on the correct classification of the type of corrugated board among the types of imperfections taken into account. The obtained results also show that the trained network models coped better with classifying five-layer samples than three-layer samples. It is unclear why the trained models classified the sample with flute E as flute EE and flute BC as flute C. However, one can notice that there was only one case of wrongly classified flutes for each of these two corrugated board types within all datasets.

In the previous study, an identification of geometrical features was proposed based on images and genetic algorithms [35,36]. These identified parameters can be used as the inputs for a simple feedforwad neural network and compared with the current approach. This was performed within a conference paper [37]. The presented comparison clearly shows that the approach based on the CNNs gives better results in comparison with the algorithm based on classical image processing operations and genetic algorithms (accuracy of 99.4% vs. 98.3%).

## 5. Conclusions

This study demonstrates the effective use of convolutional neural networks (CNNs) for classifying various types of corrugated board, a critical component in the packaging industry. The research highlights the potential of CNNs in accurately identifying flute types of corrugated boards, essential for determining their mechanical properties. The methodology showed high accuracy in classifying even deformed samples, indicating the robustness of the approach. These findings are significant for quality control and defect detection in corrugated board production, potentially leading to improved product quality and reduced waste. The study also opens up avenues for future research in applying machine learning to broader material quality assessment, extending beyond the confines of packaging. This innovative application of CNNs showcases a significant advancement in the intersection of material science and machine learning, providing a new perspective on automated quality control in manufacturing.

Furthermore, the methodology presented is the first step towards the automatic modeling of corrugated board structures. It is proven in this paper that the application of deep learning techniques in image recognition systems can evolve and help us to more accurately identify and analyze the unique characteristics of corrugated board structures from images. This involves the development of specialized algorithms that can discern subtle variances in flute size, wall construction, and paper quality. As is shown, the use of CNNs plays a pivotal role in this, enabling the systems to learn from a vast dataset of corrugated board images and improve their accuracy over time. Additionally, the incorporation of 3D modeling techniques will allow for the creation of detailed digital twins of these structures, providing invaluable insights for quality control, structural analysis, and design optimization. This progress in image recognition and modeling technology holds the promise of significant efficiency gains in manufacturing processes, quality assurance, and product development in the corrugated board industry.

## Figures and Tables

**Table 1:** Number of the corrugated board samples used to generate the dataset.

Flute Type	Number of Non-Deformed Samples	Number of Manually Deformed Samples	Number of Samples Deformed Using Creasing Machine	Total Number of Samples
flute B	48	48	48	144
flute BC	40	40	40	120
flute C	28	28	28	84
flute E	36	36	36	108
flute EB	44	44	44	132
flute EC	8	8	8	24
flute EE	12	12	12	36
Total number of samples	216	216	216	648

**Table 2:** Number of corrugated board images used in the dataset.

Class	Number of Non-Deformed Samples Images	Number of Manually Deformed Samples Images	Number of Samples Deformed Using Creasing Machine	Total Number of Images
class B	70	70	70	210
class BC	70	70	70	210
class C	70	70	70	210
class E	70	70	70	210
class EB	70	70	70	210
class EC	70	70	70	210
class EE	70	70	70	210
Total number of samples	490	490	490	1470
class Not	210			1680

**Table 3:** Accuracy results obtained for studied structures of the convolutional neural networks.

CNN Model	Accuracy (%)				Average Accuracy (%)
	*dataset1*	*dataset2*	*dataset3*	*dataset4*	
*6conv_0dense_128nodes*	98.85	98.85	98.47	100.00	99.04
*6conv_1dense_128nodes*	98.09	99.24	97.71	99.62	98.67
*5conv_0dense_32nodes*	96.95	99.24	97.71	98.85	98.19
*5conv_0dense_128nodes*	96.56	99.24	99.24	97.71	98.19
*6conv_0dense_64nodes*	96.56	98.09	98.47	99.24	98.09
*5conv_1dense_128nodes*	96.18	97.71	98.47	99.62	98.00
*5conv_1dense_64nodes*	96.56	97.71	98.47	98.85	97.90
*6conv_0dense_32nodes*	97.71	96.56	98.09	98.85	97.80
*4conv_0dense_128nodes*	96.18	96.56	97.33	98.85	97.23
*5conv_0dense_64nodes*	96.18	98.09	94.66	99.62	97.14
*6conv_1dense_64nodes*	98.09	93.51	97.71	98.47	96.95
*6conv_2dense_128nodes*	97.33	92.75	98.09	98.85	96.76
*4conv_0dense_64nodes*	95.42	98.47	95.80	97.33	96.76
*5conv_2dense_128nodes*	95.04	96.95	96.56	97.33	96.47
*4conv_1dense_128nodes*	93.51	97.71	95.04	96.95	95.80
*5conv_1dense_32nodes*	89.69	97.33	95.80	98.09	95.23
*4conv_1dense_64nodes*	92.37	97.33	94.27	96.56	95.13
*5conv_2dense_64nodes*	93.51	93.89	93.89	98.09	94.85
*6conv_2dense_64nodes*	90.08	96.56	95.80	96.16	94.65
*6conv_1dense_32nodes*	95.42	93.51	96.56	91.22	94.18
*4conv_0dense_32nodes*	92.75	93.89	92.75	96.56	93.99
*4conv_2dense_128nodes*	93.13	95.42	92.75	94.27	93.89
*4conv_2dense_64nodes*	93.51	91.98	91.60	92.75	92.46
*4conv_1dense_32nodes*	91.22	91.60	90.84	95.42	92.27
*5conv_2dense_32nodes*	90.46	91.22	88.55	92.37	90.65
*6conv_2dense_32nodes*	87.79	80.92	87.79	88.55	86.26
*4conv_2dense_32nodes*	86.64	79.77	89.69	85.11	85.30

## Data Availability

Data available on request.
